# Genetic ablation of the Bsx homeodomain transcription factor in zebrafish: Impact on mature pineal gland morphology and circadian behavior

**DOI:** 10.1111/jpi.12795

**Published:** 2022-03-31

**Authors:** Mikkel Bloss Carstensen, Adar Medvetzky, Alon Weinberger, Wolfgang Driever, Yoav Gothilf, Martin Fredensborg Rath

**Affiliations:** ^1^ Department of Neuroscience, Faculty of Health and Medical Sciences University of Copenhagen Copenhagen Denmark; ^2^ School of Neurobiology, Biochemistry and Biophysics, Faculty of Life Sciences Tel Aviv University Tel Aviv Israel; ^3^ Developmental Biology, Institute Biology, Faculty of Biology Albert Ludwig University of Freiburg Freiburg Germany; ^4^ Sagol School of Neuroscience Tel Aviv University Tel Aviv Israel

**Keywords:** *bsx*, circadian, homeobox, locomotor activity assay, loss‐of‐function, pineal gland, zebrafish

## Abstract

The pineal gland is a neuroendocrine structure in the brain, which produces and secretes the hormone melatonin at nighttime and is considered a key element in the circadian clock system. Early morphogenesis of the gland is controlled by a number of transcription factors, some of which remain active in adult life. One of these is the brain‐specific homeobox (Bsx), a highly conserved homeodomain transcription factor with a developmental role in the pineal gland of several species, including zebrafish, and regulatory roles in mature pinealocytes of the rat. To determine the role of Bsx in circadian biology, we here examined the effects of a *bsx* loss‐of‐function mutation on the pineal gland in adult zebrafish and on behavioral circadian rhythms in larvae. In pineal cell type‐specific Gfp/Egfp reporter zebrafish lines, we did not detect fluorescence signals in the pineal area of homozygous (*bsx* ^−/−^) mutants. Interestingly, a nonpigmented area on the dorsal surface of the head above the gland, known as the pineal window, was pigmented in the homozygous mutants. Furthermore, a structure corresponding to the pineal gland was not detectable in the midline of the adult brain in histological sections analyzed by Nissl staining and S‐antigen immunohistochemistry. Moreover, the levels of pineal transcripts were greatly reduced in *bsx* ^−/−^ mutants, as revealed by quantitative real‐time polymerase chain reaction analysis. Notably, analysis of locomotor activity at the larval stage revealed altered circadian rhythmicity in the *bsx* mutants with periods and phases similar to wildtype, but severely reduced amplitudes in locomotor activity patterns. Thus, Bsx is essential for full development of the pineal gland, with its absence resulting in a phenotype of morphological pineal gland ablation and disrupted circadian behavior.

## 1 INTRODUCTION

Circadian rhythms are endogenous oscillations with a period of approximately 24 h which allow animals to synchronize physiology and behavior to their surroundings; in vertebrates, the neuroendocrine hormone melatonin is produced in the pineal gland during nighttime only.^1^ The brain‐specific homeobox gene (*Bsx*), which encodes a conserved homeodomain transcription factor, seems to have functional implications in both early development of the pineal gland and mature phenotype maintenance.^2^, ^3^, ^4^, ^5^, ^6^ *Bsx* expression begins at very early stages in life and was first shown to be essential for proper pineal morphogenesis in mice.^2^, ^7^ A study on *Bsx* in the rat revealed persistent expression into adulthood specifically in the pineal gland and hypothalamus with a circadian expression pattern in the pineal gland under the control of sympathetic adrenergic signaling; *BSX* mRNA has also been detected in the human pineal gland.^6^ The study further established a role for *Bsx* in regulating transcription of other homeobox genes and genes involved in developmental processes in mature melatonin‐proficient pinealocytes.^6^

In the teleost zebrafish, which is diurnal in contrast to rodents commonly used in biomedical research, the pineal gland contains several distinct cell types including photoreceptors, neurons and glial cells,^8^ and together with the adjacent parapineal organ forms the pineal complex, the development of which is governed by a number of different transcription factors and signaling molecules.^9^, ^10^, ^11^, ^12^, ^13^, ^14^, ^15^, ^16^, ^17^, ^18^, ^19^, ^20^ Expression of *bsx* starts during the first day of development in zebrafish, before the presence of a proper pineal complex and is detectable in the majority of the pineal cell types.^5^ Knockout of *bsx* leads to aberrant morphogenesis of the pineal complex, with right‐isomerized habenulae and loss of the parapineal organ, and decreased transcript levels of prominent markers, including the enzymes tryptophan hydroxylase 1a (Tph1a), and arylalkylamine N‐acetyltransferase 2 (Aanat2), which, along with acetylserotonin O‐methyltransferase (Asmt), are responsible for the synthesis of melatonin.^5^ By use of morpholino knockdown at larval stages, a role for *bsx* in the specification of photoreceptor cell fate was further established, as had earlier been suggested from observations in *Xenopus* frogs.^4^, ^21^

However, the role of *bsx* in the pineal gland of the zebrafish has so far only been examined at embryonic and early larval stages of development. Since a number of homeobox genes have been recently shown to regulate postnatal circadian function of the mammalian pineal gland,^22^, ^23^ our aim in this study was to determine the role of *bsx* in pineal function of adult zebrafish and in behavioral circadian rhythmicity.

## 2 MATERIALS AND METHODS

### 2.1 Animals

Adult zebrafish were housed in tanks with continuous water flow at a constant temperature of 28°C on a regular 12 h:12 h light:dark schedule (12L:12D) using daylight fluorescent tubes. Light intensity was in the range of 150–500 lux depending on the location of the fish tanks (measured using an LI‐180 spectrometer, LI‐COR). For genotyping and fluorescence microscopy of adults (3.5–15 months of age), fish were anesthetized with tricaine (MS‐222). For histology and reverse transcription quantitative real‐timepolymerase chain reaction (qRT‐PCR), fish were killed by overdose with tricaine. A line of heterozygote *bsx* ^m1376^ (*bsx* ^+/^^−^) zebrafish generated in an ABTL strain was incrossed to obtain homozygous (*bsx* ^−/−^) mutants; the mutant *bsx* allele comprises a functional knockout mutation in the form of an indel region resulting in truncated mRNA lacking most of the homeodomain.^5^ The *bsx* fish line was outcrossed to the fluorescence reporter lines *foxd3:gfp* ^zf15^,^5^, ^24^ *aanat2:egfp*,^25^ and *agrp2:egfp*,^26^ and offspring were raised and PCR‐genotyped for the identification of heterozygous (*bsx* ^+/^^−^) fish. Incross of heterozygote (*bsx* ^+/^^−^) fish, one also expressing a fluorescent marker, yielded wildtype (*bsx* ^+/+^), heterozygote (*bsx* ^+/^^−^), and mutant (*bsx* ^−^^/^^−^) offspring with a Mendelian distribution. These fish were raised to adult stage, PCR‐genotyped, and photographed in vivo by fluorescence microscopy.

For histology, adult mutant (*bsx* ^−/−^) fish (approximately 12 months of age) and their wildtype (*bsx* ^+/+^) and heterozygote (*bsx* ^+/^^−^) siblings were killed during daytime and decapitated; the heads were further cut horizontally at the lower jaw and fixed in 4% PFA. For qRT‐PCR, eyes and pineal region tissue from adult (3.5–10 months of age) wildtype (*bsx* ^+/+^), heterozygote (*bsx* ^+/^^−^), and homozygous (*bsx* ^−/−^) mutant fish (total 33 females, 39 males) were dissected at Zeitgeber time (ZT) 6 (±1 h) and ZT18 (±1 h). For each genotype and tissue type at each time point, tissue from three fish was pooled into one sample, for a total of four samples per group. *foxd3:gfp* + fish were used for aided visualization of pineal tissue including parts of the skull to which the pineal complex remains attached. All animal experiments were approved by the Tel Aviv University Animal Care Committee and conducted in accordance with the requirements of the Council for Experimentation on Animal Subjects, Ministry of Health, Israel.

### 2.2 Genotyping

Genotyping was performed on a small clipping of the caudal fin from adults or on whole larvae. Samples were lysed with Proteinase K and targets of interest amplified by PCR^5^ using PCRBIO HS Taq Mix Red (PCR Biosystems) (Table S1). Genotypes were identified by agarose gel electrophoresis after enzymatic digestion of the *bsx* PCR fragment with XhoI (New England Biolabs) as previously described.^5^

### 2.3 Histology

Adult zebrafish heads were immersion fixed in 4% PFA in PBS for 2 days and stored in PBS. Heads were cryoprotected in 25% sucrose for 1 day, frozen in crushed solid CO_2_, embedded in Tissue‐Tek (Sakura Finete), and cut in 16 µm sagittal cryostat sections mounted on slides; presence of a pineal gland at any plane was verified during sectioning. To analyze brain and retinal morphology, sections of heads and eyes were stained in cresyl violet.^27^ For immunohistochemistry,^28^ sections were incubated with a rabbit polyclonal antibody against S‐antigen^29^, ^30^ (NEI Z‐02) diluted 1:500 for 44 h followed by incubation in Alexa Fluor 568‐conjugated goat anti‐rabbit IgG (Molecular Probes; catalog number A11011) diluted 1:400 for 2 h. Brightness and contrast were manually adjusted in Adobe Photoshop 7.0 (Adobe Systems Software).

### 2.4 qRT‐PCR

Total RNA was isolated by use of the RNeasy Lipid Tissue Mini Kit (Qiagen) and DNAse‐treated by use of the RNase‐Free DNase Set (Qiagen) according to the manufacturer's instructions. Three hundred and fifty nanogram of RNA was used for synthesis of cDNA with Superscript III (Invitrogen). PCR reactions were run in a Lightcycler 96 (Roche Diagnostics) at volumes of 10 µl containing 0.5 µM primers specific to the transcript of interest (Table 1), 0.2 µl cDNA and Faststart Essential DNA Green Master (Roche), on the following program: 10 min at 95°C; 40 cycles of 10 s at 95°C, 10 s at 63°C, 15 s at 72°C. Product specificity was initially confirmed by melting curve analysis and gel electrophoresis. Standard curves were generated with 10‐fold serial dilutions of pUC57 plasmids containing the target sequence (Genscript). Copy numbers were normalized against the geometric means of copy numbers of the reference genes *eef1a1* and *actb2*. All samples and standards were measured in duplicates; cutoff for positive detection was set at *C*_q_ < 32 in both duplicates of a given sample, samples above this threshold were given the value 0.

**Table 1:** Quantitative real‐timepolymerase chain reaction (qRT‐PCR) primer sequences

Transcript	Genbank acc. #	Position	Forward primer sequence (5′–3′)	Reverse primer sequence (5′–3′)
*aanat2*	NM_131411.2	200–392	ACGTTACCGGCCAGCGAGTTT	TGATGAAGGCCACCAGCTGCC
*actb2*	NM_181601.5	32–221	CACACGCAGCTAGTGCGGAA	GCGACCCACGATGGATGGGAA
*agrp2*	NM_001271291.1	176–305	ACGTGGCGCACCAAGGATCT	TCGGGCCTGGGCTTAGGTCT
*bsx*	NM_214727.1	218–412	TTGCCTCCCGGATGCCTCTTT	TTCTGGCCTTTCTGCGTCTGC
*crx*	NM_152940.1	464–553	CGCCTCAGGAATGGACCTGC	GTGCGAGTGAAGGTGGTGCG
*eef1a1*	NM_131263.1	15–135	TACCTGGCAAAGGGGAGCAGC	ACTTTCCGGAGTCGACGTGGC
*exorh*	NM_131212.2	860–1036	TCTTCGCTAACCAGGGCGCA	TGCTCTCATCCTCGGCCAGC
*otx5*	NM_181331.3	258–330	ACCGGGATGGATCTCCTGCACT	TGGTTCTTTCGCGCCGCTGT
*rho*	NM_131084.1	613–805	CTGGTGGGCTGGTCGCGTTA	CGTGCCGCCTCCTTCACAGT

### 2.5 Locomotor activity assay

Larvae from incrosses of heterozygote (*bsx* ^+/^^−^) fish were raised on a 12L:12D schedule in a 28°C incubator and at 4 days postfertilization (dpf) were individually housed in wells in 48‐well plates placed in DanioVision Observation Chambers (Noldus Information Technology). The larvae were acclimatized to the chamber for 2 days in 12 h light (white LED, 1.8 W/m^2^):12 h dim light (0.013 W/m^2^), after which their locomotor activity was monitored and tracked by EthoVision 15.0 Software (Noldus Information Technology) for 3 days from 6 to 8 dpf in constant dim light (DimDim, 0.013 W/m^2^). Raw data were converted into total distance moved (cm) in 10 min intervals throughout the 3 days of data acquisition. Following completion of the behavioral locomotor activity assay, larvae were killed by freezing, lysed whole, and genotyped.

### 2.6 Statistical analyses

Standard statistical analyses were performed using GraphPad Prism 9.00 (GraphPad Software). For qRT‐PCR, effects of genotype and ZT on mRNA levels were analyzed by two‐way ANOVA. Following two‐way ANOVA, differences for heterozygote (*bsx* ^+/^^−^) and mutant (*bsx* ^−/−^) fish compared to those of wildtype (*bsx* ^+/+^) controls were determined by multiplicity‐adjusted Dunnett's multiple comparisons tests. For the analysis of rhythmic locomotor activity, the data were normalized, and the period, phase, and amplitude were calculated as recently described.^31^ Statistical differences in period and amplitude between groups were determined by ANOVA (followed by Tukey's multiple comparisons tests), and statistical differences in phase were determined by Watson–Williams test for the homogeneity of means by use of R software. Values are presented as mean with standard error (SEM). *n*‐Values are given in the figure legends.

## 3 RESULTS

### 3.1 Pineal reporters and the pineal window are absent in adult *bsx* ^−/−^ mutant zebrafish

**Figure 1:**
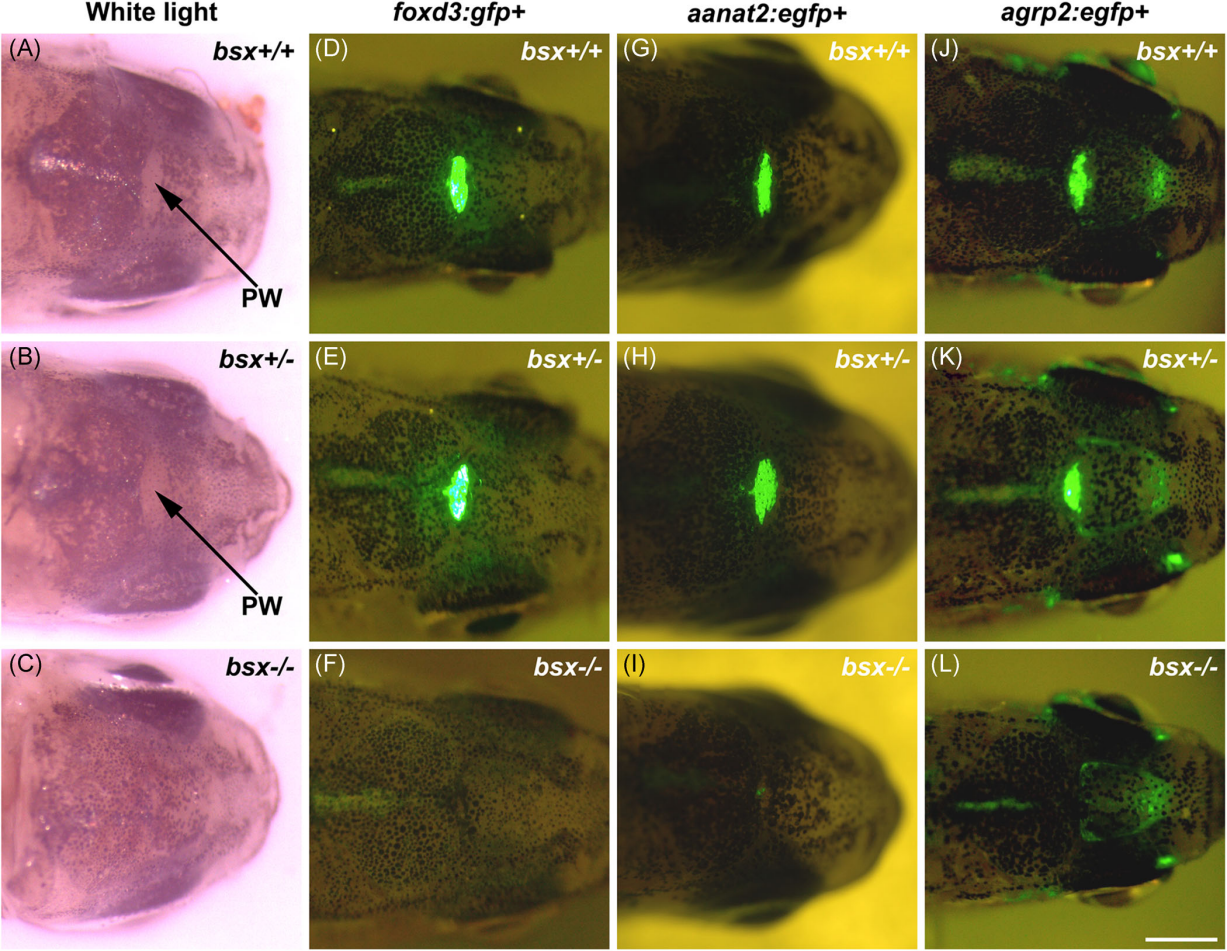
The pineal window and pineal marker signals are absent in adult *bsx* ^−/−^ mutant zebrafish. Representative light microscopy images of the pineal window (A–C) and in vivo fluorescence microscopy images of *foxd3:gfp*+ (D–F), *aanat2:egfp*+ (G–I), and *agrp2:egfp*+ (J–L) expression in adult wildtype (*bsx* ^+/+^) (A, D, G, J), heterozygote (*bsx* ^+/^^−^) (B, E, H, K) and homozygous *bsx* ^−/−^ mutant (C, F, I, L) zebrafish. Each column shows respective siblings from heterozygous incrosses; all fish were heterozygous for *gfp* or *egfp*. Scale bar, 1 mm. PW, pineal window

To establish the role of *bsx* in the mature pineal gland, incrosses of heterozygote (*bsx* ^+/^^−^) fish were raised to adulthood. Adult (3.5–15 months of age) mutant (*bsx* ^−/−^) zebrafish did not display any gross morphological abnormalities; however, the pineal window, a translucent, nonpigmented region on the dorsal surface of the head above the gland, was absent. Instead, this region was covered with pigmented cells (Figure 1A–C), as previously reported to occur in larvae after *bsx* morpholino knockdown.^21^

To facilitate identification of the pineal gland in vivo, the *bsx* mutant line was crossed with reporter fish lines expressing *gfp* or *egfp* under the control of pineal cell type‐specific promoters: forkhead box D3 (*foxd3*) for projection neurons, parapineal cells, and cone‐like photoreceptors; *aanat2* for melatonin‐producing photoreceptors; and agouti‐related protein 2 (*agrp2*) for retinal pigment epithelium‐like cells (Figure 1D–L). For all three reporter lines, strong fluorescence signals from the pineal region were observed in adult wildtype (*bsx* ^+/+^) and heterozygote (*bsx* ^+/^^−^) fish (Figure 1D,E,G,H,J,K). For the *foxd3:gfp*+ and *aanat2:egfp*+ fish, the signal was highly pineal‐specific (Figure 1D,E,G,H). For the *agrp2:egfp*+ fish, fluorescence signals were also observed in other regions, including an area above the dorsal telencephalon,^8^ in all three genotypes (Figure 1J–L). However, for all three reporter lines, fluorescence signals were not detected in the pineal region of *bsx* ^−/−^ mutants, suggesting the absence of several major pineal cell types or downregulation of the expression from the driving promoters (Figure 1F,I,L).

### 3.2 The pineal gland structure is not detected by histology in adult *bsx* ^−/−^ mutant zebrafish

**Figure 2:**
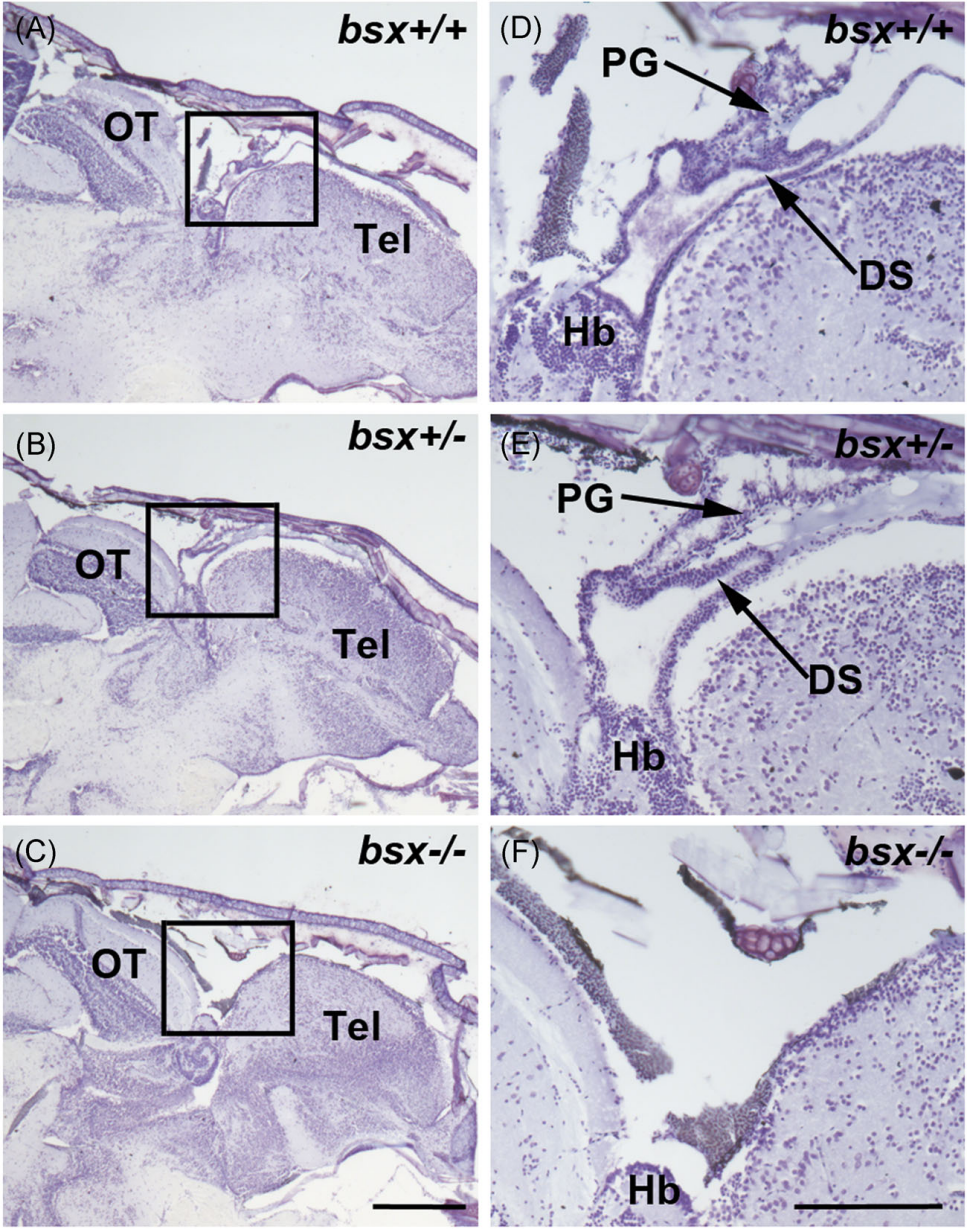
Pineal gland morphology is absent in adult *bsx* ^−/−^ mutant zebrafish. Representative images of sagittal sections of adult *bsx* ^−/−^ mutant zebrafish heads (C, F) stained in cresyl violet were examined at the midline. Images on the right (D–F) represent the boxed regions on the left (A–C) at higher magnification. Wildtype (*bsx* ^+/+^) (A, D) and heterozygote (*bsx* ^+/−^ *)* (B, E) controls are shown for comparison. For examined brains, *n* = 4 per genotype. Scale bars, 500 µm (left) and 200 µm (right). DS, dorsal sac; Hb, habenula; OT, optic tectum; PG, pineal gland; Tel, telencephalon

To determine if the absence of pineal reporter fluorescence signals in the adult *bsx* ^−/−^ mutant reflects a loss of pineal cells, the effect of *bsx* on the adult pineal gland was evaluated by performing histological analyses on adult zebrafish heads. In Nissl‐stained sagittal sections of wildtype (*bsx* ^+/+^) and heterozygote (*bsx* ^+/^^‒^) fish heads, the pineal gland was easily identified adjacent to and at the midline as a small vesicular structure attached to the dorsal roof of the skull between the pallium of the telencephalon and the optic tectum (Figure 2A,B,D,E). In homozygous (*bsx* ^−/−^) mutants, a structure corresponding to the pineal gland was not observed at any point and the pineal region was typically replaced with bone as an extension of the skull or a cavity, suggesting the total absence of the pineal gland as a result of *bsx* loss‐of‐function (Figure 2C,F). Also, the dorsal sac of the pineal complex was missing. Major morphological differences in other brain structures in the same section plane were not detected (Figure 2). In Nissl‐stained sections of eyes, morphological differences between genotypes were not observed, indicating that *bsx* is not involved in retinal formation (Figure S1).

**Figure 3:**
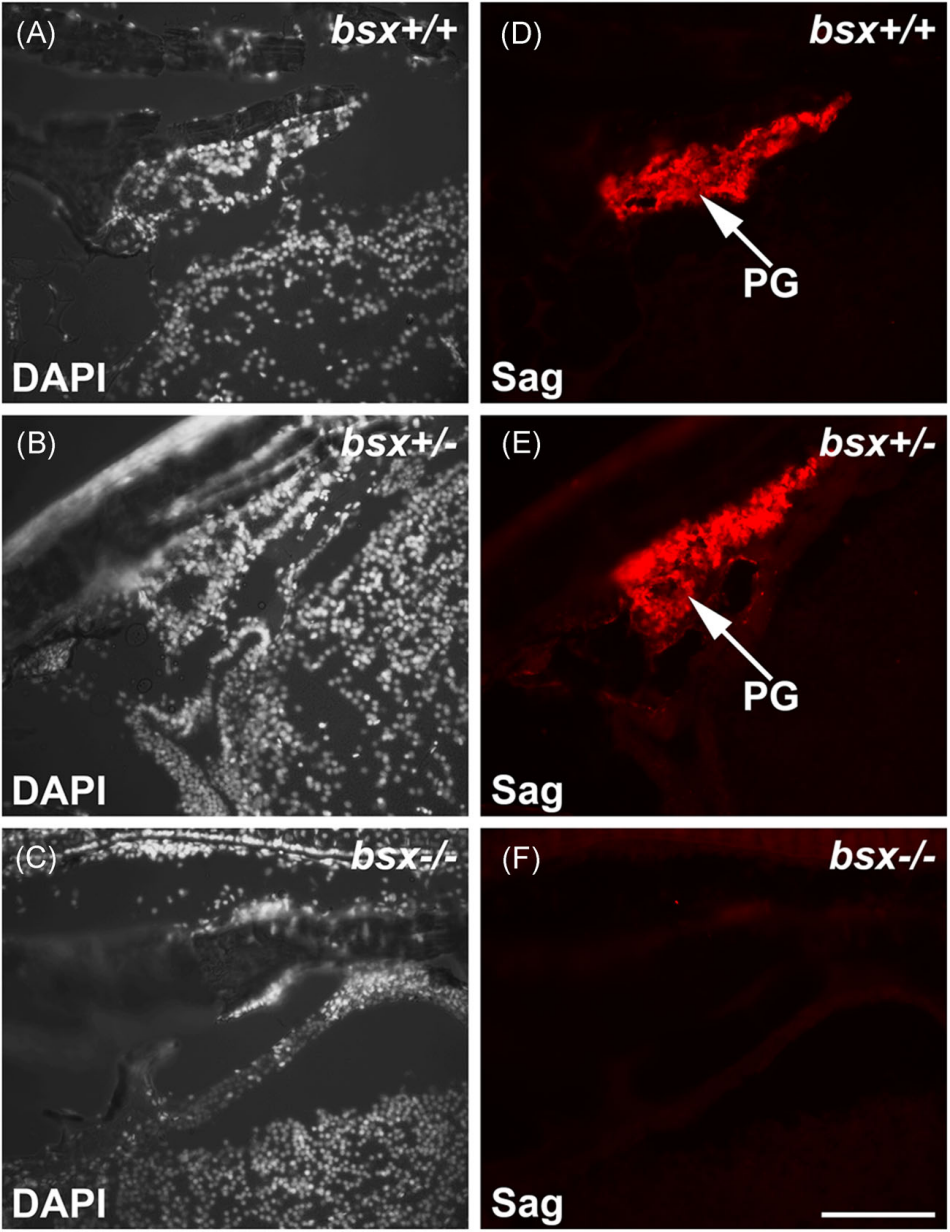
Pineal S‐antigen protein is not detectable in adult*bsx* ^−/−^ mutant zebrafish. Immunofluorescent staining for S‐antigen (Sag) on sagittal sections of adult *bsx* ^−/−^ mutant zebrafish heads (F) were examined at the midline; sections were also stained in DAPI (C). Wildtype (*bsx* ^+/+^) (A, D) and heterozygote (*bsx* ^+/−^) (B, E) controls are shown for comparison. For examined brains, *n* = 3 per genotype. Scale bar, 100 µm. PG, pineal gland

To identify the pineal gland tissue, immunohistochemistry was performed on sagittal sections of the zebrafish brain using an antibody against S‐antigen (Sag), a phototransduction protein found in pineal and retinal photoreceptors.^32^ In the brain, a strong pineal‐specific signal was detected in wildtype (*bsx* ^+/+^) and heterozygote (*bsx* ^+/^^−^) fish (Figure 3D,E), while a signal above background level was not detected in *bsx* ^−/−^ mutants (Figure 3F). In the retina, a strong, specific signal was observed in the photoreceptor nuclear layer and outer segments in all three genotypes (Figure S2), suggesting that the lack of signal in the pineal region of *bsx* ^−/−^ mutants is due to absence of the gland.

### 3.3 Pineal gland transcripts show drastically reduced levels in adult *bsx* ^−/−^ mutant zebrafish

**Figure 4:**
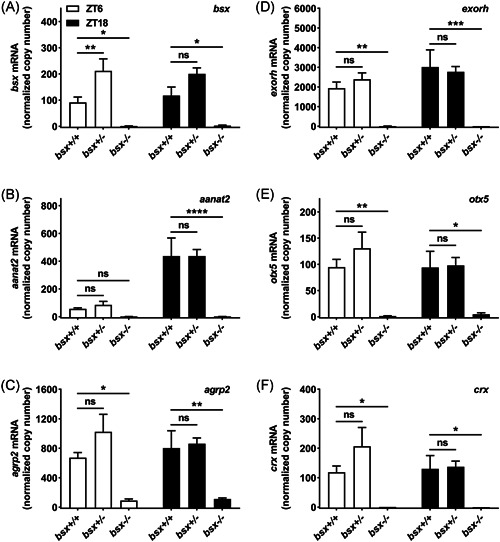
Transcript levels of pineal gland markers are greatly reduced in adult *bsx* ^−/−^ mutant zebrafish. Analyses of transcript levels of *bsx* (A) *aanat2* (B), *agrp2* (C), *exorh* (D), *otx5* (E), and *crx* (F) at daytime (ZT6) and nighttime (ZT18) as determined by qRT‐PCR. Copy numbers were normalized to the geometric means of copy numbers of *eef1a1* and *actb2*, which were detected at high and stable levels in all samples; copy numbers were further divided by 100 to account for abundant transcript levels. Two‐way ANOVA detected significant effects of time of day on transcript levels of *aanat2* (*p* < .0001) and significant effects of genotype on transcript levels of *bsx* (*p* < .0001), *aanat2* (*p* < .0001), *agrp2* (*p* < .0001), *exorh* (*p* < .0001), *otx5* (*p* < .001), and *crx* (*p* < .001). Transcript levels of homozygous (*bsx* ^−/−^) and heterozygous (*bsx* ^+/^^−^) mutants were compared to those of wildtype (*bsx* ^+/+^) controls. Values on the graphs represent mean± SEM; *n* = 4, where each sample is pooled tissue from three animals. *p*‐values as determined by multiplicity‐adjusted Dunnett's multiple comparisons tests: ns, not significant, *p* > .05; *, *p* < .05; **, *p* < .01; ***, *p* < .001; ****, *p* < .0001. ZT, Zeitgeber time

To determine levels and possible daily changes of pineal gene expression in the *bsx* ^−/−^ mutants, tissue of the pineal region was dissected from adults of all three genotypes at mid‐day (ZT6) and mid‐night (ZT18) and analyzed for transcript levels for *bsx* and the pineal markers *aanat2*,^33^ *agrp2*,^26^ extra‐ocular rhodopsin (*exorh*),^34^ orthodenticle homeobox 5 (*otx5*),^35^ and cone‐rod homeobox (*crx*)^36^ by qRT‐PCR (Figure 4).

Analysis of *bsx* mRNA levels revealed a significant effect of genotype by two‐way ANOVA (*p* < .0001), but not for time of day (*p* > .05). Levels were greatly decreased in *bsx* ^−/−^ mutants (*C*_q_ > 32 in four of eight samples) compared to wildtype (*bsx* ^+/+^) controls at both time points (*p* < .05, Dunnett's multiple comparisons test). As the primer pair targets a region of the mRNA before the truncation, this does not distinguish between wildtype and mutant transcripts, but strongly suggests the absence of *bsx*‐expressing cells in samples from *bsx* ^−/−^ mutants (Figure 4A). Increased levels of the *bsx* transcripts were detected in heterozygotes (*bsx* ^+/^^−^) (*p* < .01, Dunnett's multiple comparisons test) (Figure 4A).

Two‐way ANOVA revealed significant effects of genotype on *aanat2* (*p* < .0001), *agrp2* (*p* < .0001), *exorh* (*p* < .0001), *otx5* (*p* < .001), and *crx* (*p* < .001) mRNA levels (Figure 4B–F). All five transcripts were reduced to very low levels in the *bsx* ^−/−^ mutants, with significant changes compared to wildtype (*bsx* ^+/+^) controls as determined by Dunnett's multiple comparisons tests: *aanat2* (ZT18, *p* < .0001), *agrp2* (ZT6, *p* < .05; ZT18, *p* < .01), *exorh* (ZT6, *p* < .01; ZT18, *p* < .001), *otx5* (ZT6, *p* < .01; ZT18, *p* < .05), and *crx* (*p* < .05; *C_q_* > 32 in seven of eight samples) (Figure 4B–F). A significant effect of time of day was detected for *aanat2* (*p* < .0001; two‐way ANOVA) with higher expression levels at nighttime, reflecting the nocturnal increase of *aanat2* levels resulting in melatonin production at nighttime (Figure 4B).^33^ Significant effects of ZT were not detected for *agrp2, exorh, otx5*, and *crx* (*p* > .05; two‐way ANOVA), indicating no day–night differences for these transcripts at the examined time points (Figure 4C–F). For the examined pineal markers, levels were unchanged between wildtype (*bsx* ^+/+^) and heterozygote (*bsx* ^+/^^−^) zebrafish (*p* > .05; Dunnett's multiple comparisons test), suggesting haplosufficiency of *bsx* (Figure 4).

qRT‐PCR analysis of whole eyes did not reveal *bsx* mRNA at detectable levels in any of the genotypes (*C_q_* > 32 for all but 5 of 24 samples), and transcript levels of the phototransduction gene rhodopsin (*rho*)^37^ did not differ between genotypes and time points (*p* > .05; two‐way ANOVA and Dunnett's multiple comparisons test) (Figure S3).

### 3.4 Rhythmic behavior is disrupted in *bsx* ^−/−^ mutant zebrafish larvae

**Figure 5:**
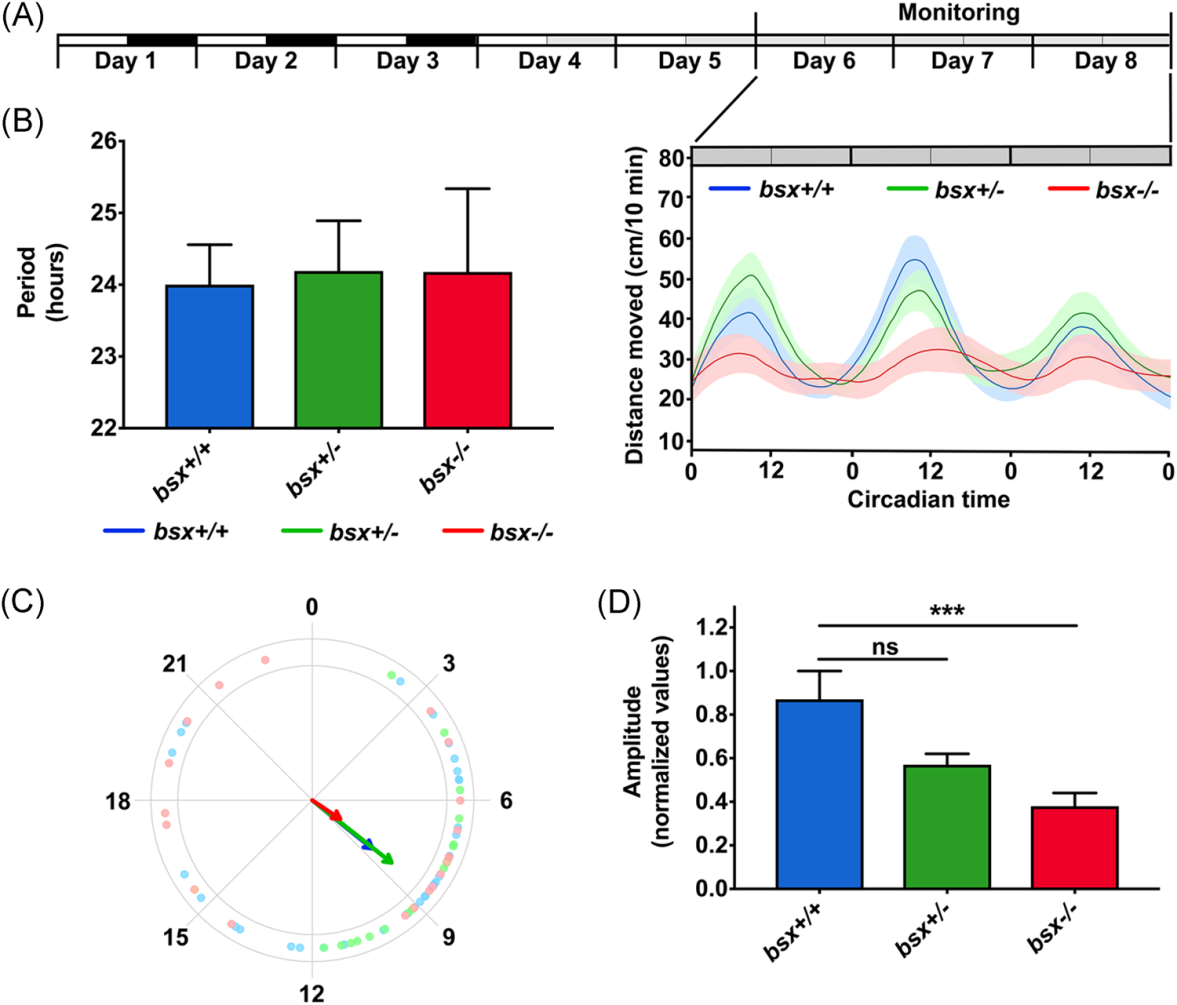
Behavioral rhythms of locomotor activity are disrupted in *bsx* mutant zebrafish larvae. (A) Locomotor activity of WT (blue, *n* = 20), *bsx* ^+/^^−^ (green, *n* = 38) *and bsx* ^−/−^ (red, *n* = 20) zebrafish larvae monitored under a constant dim light schedule (DimDim) from 6 to 8 dpf, after entrainment by three 1 L:12D cycles and two light‐dim light cycles. The average distance moved (cm/10 min) is plotted on the *y*‐axis and circadian time (CT) is plotted on the *x*‐axis; error bars indicate SEM. The experimental design of the photic treatment before and throughout activity monitoring is shown at the top. White boxes represent light, black boxes represent dark, and gray boxes represent dim light. (B) Bar graphs displaying average periods of the three genotypes. No significant differences were detected by ANOVA (*p* > .05). (C) Circular plots of the average circadian phase of the rhythms of the three genotypes. Arrow directions represent the average phase for each genotype and arrow length represents the variance (longer arrow stands for low variance and vice versa); dots represent the values of individual larvae (note the high variance in *bsx* ^−/−^ mutant larvae). (D) Bar graphs displaying the mean amplitudes of the relative rhythms for the three genotypes, showing significant effect of the genotype (*p* = .0013, ANOVA); mutant (*bsx* ^−/−^) larvae display a decreased amplitude compared to wildtype (*bsx* ^+/+^) larvae (*p* < .001, Tukey's multiple comparisons tests). ***p < .001; ns, not significant

To determine the effect of *bsx* deficiency on behavioral circadian rhythms, the locomotor activity of zebrafish larvae, progeny of a cross between heterozygous (*bsx* ^+/^^−^), fish was analyzed. Larvae were entrained to 12L:12D, and then their locomotor activity was tracked under DimDim conditions for 3 days from 6 to 8 dpf. The plotted average locomotor activity of wildtype (*bsx* ^+/+^), heterozygotes (*bsx* ^+/^^−^), and *bsx* ^−/−^ mutants are displayed in Figure 5A. Total locomotor activity did not differ between genotypes (*p* > .05, ANOVA). Circadian rhythms of locomotor activity were maintained in all three genotypes with no significant difference in period (Figure 5B; *p* > .05, ANOVA). The average phases also did not differ between genotypes (Figure 5C; *p* > .05, Watson−Williams test), with an average peak of activity at CT 8.55 ± 0.66 for wildtype (*bsx* ^+/+^), CT 8.6 ± 0.66 for heterozygotes (*bsx* ^+/^^−^) and CT 8.26 ± 1.42 for *bsx* ^−/−^ mutants (note the higher variability in *bsx* ^−/−^ mutant fish). Notably, a substantial difference in relative amplitudes between genotypes was detected (Figure 5D; *p* < .01, ANOVA). Reduced amplitude of locomotor activity circadian rhythms, without an effect on the period or phase, was previously observed in melatonin‐deficient zebrafish^38^ and in zebrafish in which the molecular circadian oscillator was blocked specifically in the melatonin‐producing pineal photoreceptor cells.^39^ Thus, loss of *bsx* function, likely via the loss of the pineal gland, leads to disruption of circadian locomotor activity rhythms, by significantly reducing the amplitude of the rhythm.

## 4 DISCUSSION

This study establishes that the Bsx homeodomain transcription factor is essential for function of the pineal gland in adult zebrafish and consequently contributes to maintaining behavioral circadian rhythms in zebrafish larvae. Bsx in the pineal gland has been previously reported in terms of its developmental role during early life of mice, *Xenopus* frogs, and zebrafish, and its regulatory role in adult rats.^4^, ^5^, ^6^, ^7^ We here expand current knowledge of Bsx by reporting a morphological absence of the pineal gland and greatly reduced levels of pineal transcripts in adult *bsx* ^−/−^ zebrafish mutants through multiple approaches and establish a role of *bsx* in circadian biology. Our data support the concept that *Bsx* across species is essential for development of the pineal gland in early life and that loss of Bsx function results in a distinct phenotype persisting into adulthood.

The zebrafish pineal gland is a heterogeneous structure composed of several different cell types expressing *bsx* ^5^ with single RNA sequencing data suggesting expression in pineal photoreceptors and neurons.^8^ The morphological absence of the pineal gland in *bsx* ^−/−^ zebrafish mutants was accompanied by loss of the pineal window and the dorsal sac. We speculate that the presence of the pineal gland may be required to induce formation or maintenance of the pineal window of the skin, whereas the dorsal sac, in which the expression of an arylalkylamine N‐acetyltransferase isoform (*aanat1b*) has been reported,^40^ may in line with the pineal gland itself require endogenous *bsx*. Further, our analyses of transcript levels of markers for specific pineal cell types revealed greatly reduced levels for all examined transcripts, often several hundred‐fold. One of the transcripts, namely *agrp2*, a marker for pineal retinal pigment epithelium‐like cells,^26^ was detectable within the threshold, though at very low numbers, potentially reflecting extra‐pineal expression of this gene.^8^, ^26^ Other examined transcripts, such as *crx*, seem to be completely absent, while S‐antigen protein was undetectable by immunohistochemistry, supporting the notion that pineal photoreceptors are absent and that the main function of the pineal gland, namely maintaining circadian rhythms, is disrupted by the *bsx* loss‐of‐function mutation.

The Bsx protein exerts its function as a transcription factor.^3^, ^21^ However, since a fully formed gland was not present following the *bsx* loss‐of‐function mutation, as evidenced by other methodological approaches, it was not possible to conclude on a potential role in transcriptional regulation of other genes in the mature zebrafish pineal gland; markedly reduced transcript levels are most likely reflective of the absence of pineal cells rather than downregulated gene expression. Unexpectedly, increased levels of the *bsx* transcript were detected in the pineal area of heterozygotes (*bsx* ^+/−^) fish; this may reflect a compensatory mechanism. In the rat, limitations of a knockout approach were recently overcome by employing siRNA‐mediated knockdown in cell cultures of mature pinealocytes from fully formed glands from adults; in this setup, BSX was shown to control two other homeobox genes, *Pax4* and *Otx2*, as well as genes mapped to developmental processes, but not those encoding the melatonin‐synthesizing enzymes.^6^ This points to an intrinsic regulation mechanism for homeobox genes in the pineal gland, further evidenced by mutual regulation of *Crx* and *Otx2*.^22^, ^41^ A similar regulatory pattern occurs in zebrafish, as *otx5* functions downstream from *bsx*, while *bsx* itself functions downstream from floating head (*flh*).^5^, ^21^ In addition to mutual feedback regulation, several homeodomain transcription factors also control melatonin production in the rat,^22^, ^23^, ^27^, ^42^ while the zebrafish *aanat2* gene contains photoreceptor‐conserved elements—binding sites for homeodomain transcription factors—in the promoter region and is positively regulated by Otx5.^35^, ^43^, ^44^ Our novel finding that both *bsx* and a number of other homeobox genes are expressed in the mature zebrafish pineal gland gives reason to believe that regulatory roles of these homeodomain transcription factors also persist in the pineal gland at later stages.

The rat *Bsx* homolog exhibits a prominent circadian rhythm of expression in the pineal gland of adults with a peak in the middle of the subjective night.^6^ Similar patterns of daily changes in expression have been reported for other homeobox genes in the mature rat pineal gland, namely *Pax4*, *Lhx4*, *Rax*, *Otx2*, and *Crx*.^22^, ^23^, ^42^, ^45^, ^46^ In this study, we establish that the homeobox genes *bsx*, *otx5*, and *crx* are expressed in the mature zebrafish pineal gland; however, we did not detect day‐night differences in expression of these genes. This suggests nonrhythmicity for at least some of the homeobox genes in the pineal gland of adult zebrafish, as differences at the examined time points would be expected for the corresponding homologs in rats.^6^, ^42^ Arrhythmic expression of pineal gland *bsx* and *otx5* mRNAs was also observed at the larval stage, while *crx* has been reported to exhibit a circadian rhythm of expression at a whole‐organism level; in *Xenopus*, pineal *Xbsx* mRNA exhibits daily fluctuations at early developmental stages.^4^, ^5^, ^35^, ^47^ Together, these studies reveal different expression patterns of pineal homeobox genes in different species despite largely conserved functions, suggestively reflecting different entrainment mechanisms: In mammals, rhythmicity of the pineal gland depends on the endogenous circadian clock of the suprachiasmatic nucleus acting via the sympathetic nervous system, while pineal glands of nonmammalian vertebrates are directly photoreceptive and contain an intrinsic circadian clock.^48^, ^49^

The altered circadian rhythms in locomotor activity of the *bsx* mutant larvae, characterized by reduced amplitude, suggest an important role of this gene in circadian biology. Similarly altered behavioral rhythms have been reported in zebrafish larvae with other types of pineal dysfunction, including an *aanat2* knockout mutant and a conditional dominant‐negative mutant for the clock gene *clock* in melatonin‐producing cells.^38^, ^39^ Our data suggest that the loss of the pineal gland is the cause of the altered circadian rhythm, but since period and phase were unaffected, other brain circuits controlling circadian biology of the zebrafish, such as hypothalamic QRFP‐expressing neurons and their projections to QRFP receptor‐expressing target areas,^50^ seem to be sufficient to maintain rhythmicity. In addition to period and phase, overall activity remained at similar levels in all genotypes, reflecting the importance of other CNS regions in governing general locomotor activity.^51^ Several aspects of aberrant pineal function could result in the abnormal circadian rhythmicity of motor activity, obviously including disruptions in melatonin production, or alternatively photoreceptive capabilities and activity of projection neurons. Our histological analyses did not reveal any changes in brain morphology apart from that of the pineal gland, but dysfunction in other brain regions, namely the hypothalamus,^2^, ^3^, ^7^, ^52^, ^53^, ^54^, ^55^, ^56^ could play a role as well. However, the mutant phenotype reported here, that is, a zebrafish without a pineal gland, may prove to be an excellent model for studying pineal function in a context of circadian physiology in a diurnal species.

## AUTHOR CONTRIBUTIONS

Mikkel B. Carstensen designed experiments, performed experiments, analyzed data, and drafted the manuscript. Alon Weinberger and Adar Medvetzky performed experiments and analyzed data. Wolfgang Driever provided resources, gave advice, and edited the manuscript. Yoav Gothilf and Martin F. Rath conceived the study, designed experiments, analyzed data, and revised the manuscript. All authors approved the final manuscript.

## CONFLICTS OF INTEREST

The authors declare no conflicts of interest.

## Supporting information

Supporting information.

## Data Availability

The data that support the findings of this study are available from the corresponding author upon reasonable request.
