# Supplementary material for: Genetic ablation of the Bsx homeodomain transcription factor in zebrafish: Impact on mature pineal gland morphology and circadian behavior
Source: J Pineal Res. 2022 Mar 31;72(4):e12795. doi: 10.1111/jpi.12795 (PMC9285933; doi:10.1111/jpi.12795)
Supplement: Supplementary file 1 — Supporting information. [file JPI-72-0-s001.pdf]

SUPPLEMENTARY MATERIAL

Table S1. PCR primer sequences for genotyping

Transcript	Forward primer sequence (5´-3´)	Reverse primer sequence (5´-3´)
bsx	ATTGCAAAAGGAATGCAGATG	ATTGTCGTCCAGCGTGTATCT
gfp	TTTTCAAGAGTGCCATGCCC	CCATGTGTAATCCCAGCAGC
egfp	CAGATGAACTTCAGGGTCAG	CTGCCGTCCTCGATGTTGTG

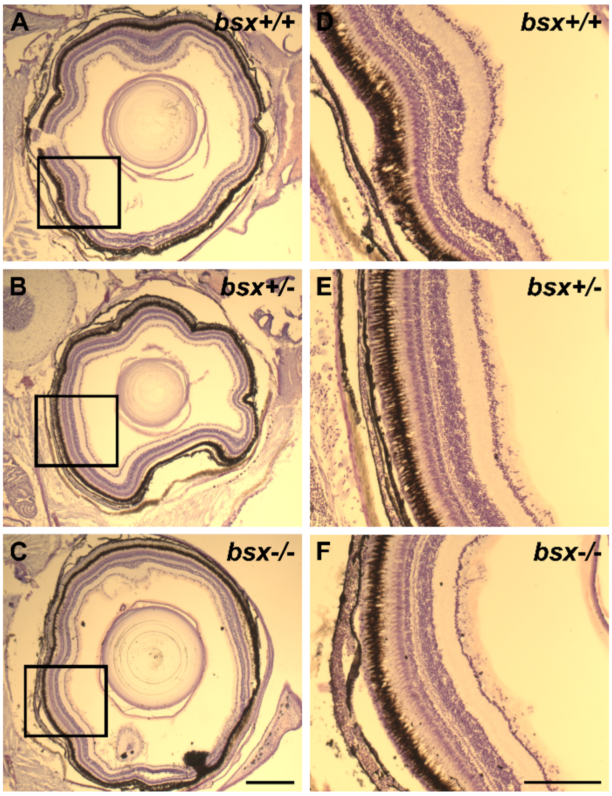

Figure S1. Morphological structure of the retina is unaffected in adult *bsx*$^{-/-}$ mutant zebrafish. Representative images of sagittal sections of *bsx*$^{-/-}$ mutant zebrafish eyes (**C, F**) stained in cresyl violet. Images on the right (**D-F**) represent the boxed regions on the left (**A-C**) at higher resolution. Wildtype (*bsx*$^{+/+}$)(**A, D**) and heterozygote (*bsx*$^{+/-}$)(**B, E**) controls are shown for comparison. For examined eyes, n=4 per genotype. Scale bars, 500 µm (left) and 200 µm (right).

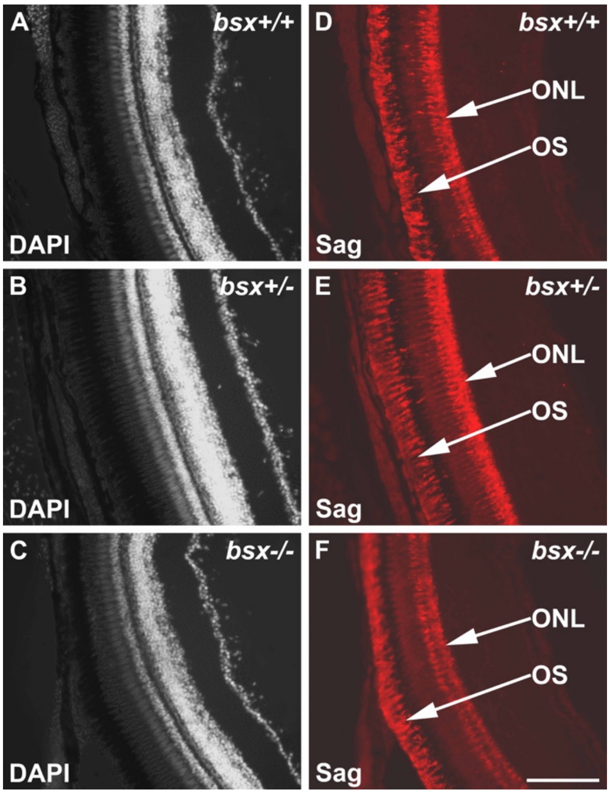

Figure S2. S-antigen protein is present in the retina of adult *bsx*$^{-/-}$ mutant zebrafish. Immunofluorescent staining for S-antigen (Sag) on sagittal sections of adult *bsx*$^{-/-}$ mutant zebrafish retina (**F**); sections were also stained in DAPI (**C**). Wildtype (*bsx*$^{+/+}$)(**A, D**) and heterozygote (*bsx*$^{+/-}$)(**B, E**) controls are shown for comparison. Scale bar, 100 µm. ONL, outer nuclear layer, OS, photoreceptor outer segments.

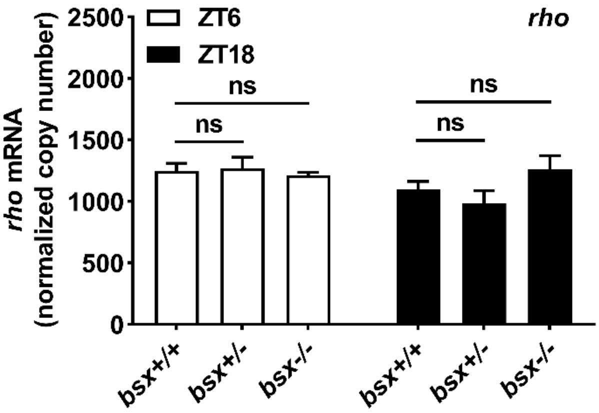

Figure S3. Abundant *rho* transcript levels are maintained in whole eyes of adult *bsx*$^{-/-}$ mutant zebrafish. Analyses of transcript levels of rhodopsin (*rho*) at daytime (ZT6) and nighttime (ZT18) as determined by qRT-PCR. Copy numbers were normalized to the geometric means of copy numbers of *eef1a1* and *actb2*, which were detected at high and stable levels in all samples; copy numbers were further divided by 10000 to account for abundance of transcripts. Two-way ANOVA did not detect significant effects of genotype or time of day. Transcript levels of homozygous (*bsx*$^{-/-}$) and heterozygous (*bsx*$^{+/-}$) mutants were compared to those of wildtype (*bsx*$^{+/+}$) controls. Values on the graphs represent mean ± SEM; n=4, where each sample is pooled tissue from 3 animals. P-values as determined by Dunnett's multiple comparisons test: ns, not significant. ZT, Zeitgeber time.
